# Enhancing Separation Performance of PA Nanofiltration Membrane Through Polyelectrolyte PSS Interlayer and Surface Modification

**DOI:** 10.3390/polym18101242

**Published:** 2026-05-19

**Authors:** Fotios Panagiotou, Georgia Zafeiropoulou, Franceska Gojda, Kiriaki Chrissopoulou, Ioannis Zuburtikudis, Valadoula Deimede

**Affiliations:** 1 Department of Chemistry, University of Patras, 26504 Patras, Greece; fpanagiotou@proton.me (F.P.); geozafiropulu@gmail.com (G.Z.); 2 Institute of Electronic Structure and Laser, Foundation for Research and Technology—Hellas, 71110 Heraklion, Greece; fgkointa@physics.uoc.gr (F.G.); kiki@iesl.forth.gr (K.C.); 3 Department of Physics, University of Crete, 70013 Heraklion, Greece; 4 Chemical Engineering Department, Abu Dhabi University, Abu Dhabi 59911, United Arab Emirates; ioannis.zuburtikudis@adu.ac.ae

**Keywords:** nanofiltration, TFC membrane, water permeability, salt rejection

## Abstract

Thin-film composite (TFC) polyamide (PA) nanofiltration membranes are the state of the art for water purification and reclamation, although a selectivity–permeability trade-off often restricts their development. To mitigate this problem, in this work, a novel three-layer structured nanofiltration (NF) membrane was fabricated consisting of a negatively charged poly (sodium 4-styrenesulfonate) (PSS) interlayer, a high-performance polyethyleneimine (PEI)-based PA separation layer and a PEI-grafted top layer. The PSS interlayer aimed to regulate interfacial polymerization (IP) of PEI with trimesoyl chloride (TMC) and enhance water transport, while PEI-grafting ensured high salt rejections. The relevant characterizations indicated that PEI-grafting endowed the resulting membrane (I-TFC-g) with a positive surface charge and increased the crosslinking degree to achieve much higher rejections for Mg^+2^ ions through the synergistic effect of Donnan and size-exclusion mechanisms, while the incorporation of the PSS interlayer resulted in an increased pure-water permeability (PWP) value of 7 L m^−2^ h^−1^ bar^−1^ (a value 2.8 times higher compared to the membrane TFC-g without a PSS interlayer). In specific, the I-TFC-g membrane displayed the highest salt rejections of 91% for MgCl_2_, 92% for MgSO_4_, 73% for Na_2_SO_4_ and 58% for NaCl and a good long-term stability. Overall, this work presents a simple strategy to improve NF performance by simultaneous enhancement of water permeability and salt selectivity.

## 1. Introduction

Having access to clean water is becoming increasingly difficult due to severe water pollution and water shortages [1,2,3]. In this context, nanofiltration (NF) is emerging as an efficient method for water reclamation and purification. Specifically, due to its low operating pressure, high permeability and ability to separate mostly divalent ions and organic pollutants, NF has been applied for water-based purifications and resource recovery [4,5,6,7,8,9].

State-of-the-art NF membranes exhibit a thin-film composite (TFC) morphology, which consists of a porous support and a thin rejection polyamide (PA) layer. The most common method for the synthesis of the thin PA layer (~100 nm) is via interfacial polymerization (IP) between an amine monomer (e.g., piperazine, PIP) and trimesoyl chloride (TMC) [10,11]. These NF membranes typically have nanopores and a negative surface charge owing to the hydrolysis of acyl chloride groups into carboxyl groups during the IP. As a result, the NF separation is mainly governed by size exclusion, Donnan exclusion, diffusion-solution and dielectric effect mechanisms [11,12,13]. Despite the big advances in separation performance of TFC polyamide NF membranes [14,15,16,17,18,19,20,21], they are still restricted by a trade-off between water permeability and salt selectivity [22,23]. Improving water permeability without sacrificing salt selectivity is crucial for the preparation of NF membranes working with lower energy consumption and/or increased production [12].

The incorporation of an interlayer between the porous substrate and the selective layer serves as an effective route to address this bottleneck via a simultaneous selectivity and water permeability enhancement. In specific, the inclusion of an interlayer can shorten the water transport pathway, thereby leading to an enhanced water permeance (e.g., gutter effect) [24], and can also result in a more uniform and thinner PA rejection layer with a reduced pore size and minimal defects (by regulating the rate and the quality of the IP reaction), thus improving both the selectivity and water permeance [25,26,27,28]. Having in mind that polymeric interlayers can be reliably and economically integrated into commercial membrane manufacturing and water treatment technologies, their successful application for improving divalent/monovalent ion separation selectivity of NF membranes has been reported. In particular, polymeric interlayers based on ionic polyelectrolytes [29,30,31,32,33], polydopamine [34,35,36], and poly(vinyl alcohol) [27,37,38,39] were used to regulate the IP reaction through storage and diffusion control of the amine monomer, thereby affecting the pore size, PA thickness and surface charge density of the NF membrane. In addition, such reactive/functional interlayers reinforced the interfacial strength between the PA rejection layer and the substrate through hydrogen bonding, electrostatic attractions and/or covalent bonding [36,37,40], thereby enhancing the long-term membrane performance stability in terms of water flux and salt rejection. Meanwhile, if the interlayer has an opposite charge property with the polyamide layer, the prepared composite membrane can achieve high rejection of divalent anions and cations simultaneously [41,42,43].

Among polymeric interlayers, the water-soluble ionic polyelectrolytes prepared by the layer-by-layer (LBL) coating method, which enables nanometer-scale layer formation, have gained significant attention recently [29,30,31,32,33]. For example, Wang et al. prepared a TCF NF membrane comprising a positively charged polyelectrolyte interlayer obtained by LBL self-assembly of chitosan/sodium alginate and a negatively charged polyamide separation layer that exhibited enhanced divalent anion/cation rejection (e.g., MgSO_4_ 88.11%, Na_2_SO_4_ 89.11%, MgCl_2_ 81.65%) and a moderate decrease in water permeability (from ~20 to 15.5 L m^−2^ h^−1^ bar^−1^) [29]. Tian et al. incorporated a sodium alginate interlayer that significantly enhanced the rejection for divalent ions compared to the control membrane (from ~40% to 91.12% for MgCl_2_), while a slight decrease in permeability was observed (from 9.71 to 7.23 L m^−2^ h^−1^ bar^−1^) [30]. He et al. introduced a PSS/PEI interlayer via LBL to regulate the IP of PIP and TMC, which led simultaneously to a water permeability increase (~15 Lm^−2^h^−1^bar^−1^), very high rejections for MgSO_4_ and Na_2_SO_4_ (~99%) and increased rejections for MgCl_2_ (70%) [31].

Another effective route to achieve a high rejection of divalent cations of PA NF membranes is to impart a positive charge on the membrane surface through post-treatment [44,45]. In specific, the unreacted acyl chloride groups remaining on the surface of the NF membrane after primary interfacial polymerization can directly react with amino-containing monomers to achieve a high amine density on the NF membrane surface [46,47]. This in turn enhances the positive charge density of the NF membrane surface, thereby improving the divalent/monovalent cation separation selectivity.

**Figure 1:**
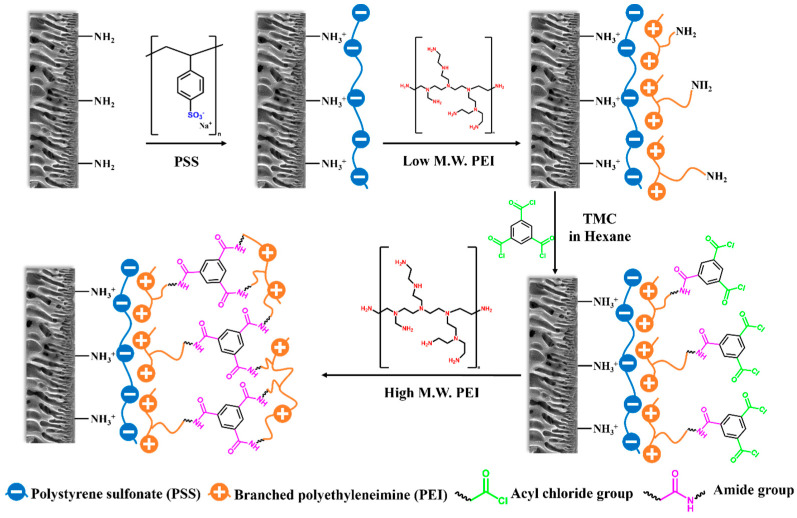
Schematic representation of the fabrication of NF membranes.

In light of this, a novel three-layer structured composite NF membrane was fabricated consisting of a negatively charged PSS interlayer, a high-performance PEI-based PA separation layer and a PEI-grafted top layer (Figure 1). In this novel approach, PEI has a dual role, serving both as the layer-by-layer polycation and as the amine monomer for IP, resulting in one-step construction of the PA layer without needing an additional PIP adsorption step. The hydrophilic PSS interlayer containing abundant sulfonate groups was deposited on porous substrate, aiming to improve the water permeance and regulate the subsequent deposition of the polycationic PEI, via electrostatic adsorption (LBL method), as an IP monomer. The interlayer is critical to the following layered IP process, as it controls the amount of PEI monomer available for the polymerization to only the amount adsorbed by the interlayer. In addition, due to the strong interaction between PEI and PSS, only a low concentration of PEI reacts with TMC during IP, thus enabling the formation of a looser PA layer with a negative surface charge due to many unreacted acyl chloride groups of TMC. To improve the membrane’s properties, PEI-grafting via a secondary IP reaction with residual TMC took place to impart a positive charge density and an improved compactness on the PA surface (Figure 1). This comprehensive modification strategy led to the development of an I-TFC-g membrane with simultaneously enhanced water permeability and salt rejections, since grafting with PEI reinforced both the size-sieving and the Donnan exclusion mechanisms, while the incorporation of the hydrophilic PSS interlayer contributed to a water permeability enhancement.

## 2. Materials and Methods

### 2.1. Materials and Chemicals

Sodium chloride (99%), sodium sulfate (99%), magnesium sulfate (99.5%), magnesium chloride anhydrous (98%), polyvinylpyrrolidone (PVP, M_w_ = 29,000 gmol^−1^), 1,3,5-benzenetricarbonyl trichloride (TMC, 98%), hexane (99%) and polysulfone (PSf, M_n_ = 26,000 gmol^−1^) were purchased from Sigma-Aldrich (Darmstadt, Germany). The solvent N-methyl-2-pyrrolidone (NMP, 99+%) and sodium polystyrene sulfonate (PSS, M_w_ = 70,000 gmol^−1^) were purchased from Alfa Aesar (Ward Hill, Massachusetts, MA, USA). Branched polyethyleneimine (PEI, M_w_ = 2000 gmol^−1^) was purchased from Thermo Fisher Scientific (Waltham, Massachusetts, MA, USA) and a higher-molecular-weight branched polyethyleneimine (M_w_= 25,000 gmol^−1^) was purchased from Sigma Aldrich (Darmstadt, Germany). Ultra-pure water (Type 3) was obtained by the Arium ^®^ Mini Plus from the Sartorius AG Company (Göttingen, Germany).

### 2.2. Composite NF Membrane Preparation

#### 2.2.1. Fabrication of S1 Porous Substrate

A PSf porous substrate with a cationic surface charge was prepared, aiming to strengthen the adsorption of anionic polyelectrolyte PSS (interlayer) via electrostatic attraction. The substrate was prepared via the non-solvent-induced phase separation (NIPS) method. Firstly, polyvinylpyrrolidone (PVP) and branched polyethyleneimine (PEI, M_w_ = 2000 gmol^−1^) were dissolved in NMP, followed by the addition of the PSf polymer in portions under vigorous stirring. The final concentrations of PVP, PEI and PSf were 5% *w*/*w*, 0.6% *w*/*w* and 15% *w*/*w*, respectively. A homogeneous dope solution was obtained after stirring for 18 h followed by degassing under a vacuum for 2 h at room temperature to remove air bubbles prior to the casting process. Subsequently, it was cast onto a glass surface using a doctor blade with a thickness set at 200 μm (Sheen, UK). The cast substrate was immediately immersed into a water coagulation bath at room temperature to initiate the phase inversion. The as-prepared, positively charged porous substrate (denoted S1) was detached from the glass surface within a few minutes, transferred to another bath containing fresh deionized water, and kept for 24 h (the solvent was changed once) to remove residual solvent. The presence of PEI in the S1 matrix was confirmed by ATR-FTIR spectroscopy (Figure S1). In particular, the peak observed at 1035 cm^−1^, corresponding to C-N stretching of PEI, suggests that PEI was successfully incorporated into the S1 substrate. The membranes were stored in an 80% glycerol/water solution to avoid porous collapse during the subsequent thermal treatments.

#### 2.2.2. Synthesis of TFC NF Membranes

The TFC NF membrane was prepared via layered interfacial polymerization combining the LBL method with IP [48] and post-modification. The S1 substrate was first coated with the anionic polyelectrolyte PSS (interlayer), followed by deposition of cationic PEI via electrostatically driven complexation using the layer-by-layer (LBL) method. The latter served as a polyamine monomer and reacted with TMC via IP for the preparation of the selective layer. Specifically, the LBL method was conducted by dip-coating only the active layer surface of the porous support. In more detail, the positively charged porous substrate S1 was fixed between an acrylic plate and a rubber frame with the active layer facing upwards. After rinsing the surface with deionized water to remove the excess of the glycerol solution (that was used on the substrate preparation), a 0.4% *w*/*v* PSS solution (3% *w*/*v* NaCl, pH = 4) was poured onto the substrate for 5 min and its excess was washed off by using 3% *w/v* NaCl to remove the non-adsorbed polyelectrolyte. Next, a 0.4% *w*/*v* PEI (M_w_ = 2000 gmol^−1^) (polycation) solution (adjusted with 3% *w*/*v* NaCl) was gently poured onto the surface for 5 min, followed by the same rinsing procedure as described previously. After the above LBL procedure, one bilayer of PSS/PEI was deposited on top of the porous support.

**Figure 2:**
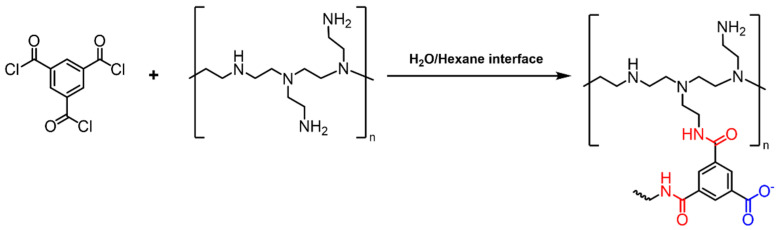
Interfacial polymerization between TMC and PEI.

Following PSS/PEI LBL deposition, the PA layer was formed via IP polymerization (Figure 2). After removing the excess water by purging with pressurized air, a 0.25% *w*/*v* TMC hexane solution was poured onto the surface for 1 min, followed by rinsing with n-hexane to remove excess TMC. The obtained membrane was dried in the oven for 6 min at 90 °C for further crosslinking. The prepared NF PA membranes were denoted as “I-TFC”. A PA membrane was prepared on S1 substrate without applying a PSS interlayer and used as a control for comparison (“TFC”).

**Figure 3:**
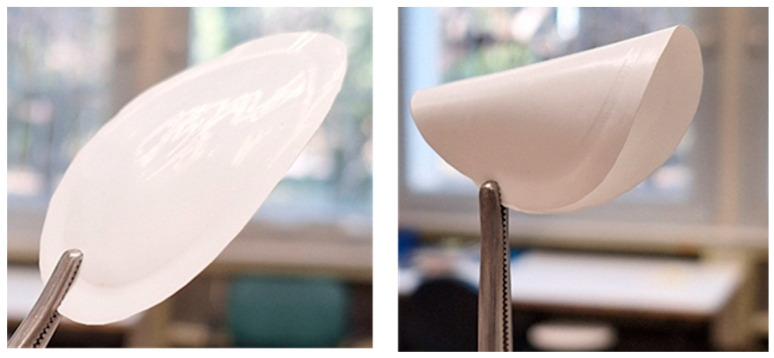
The prepared I-TFC-g membranes.

The NF membranes with the PA layer were further grafted with PEI. In particular, a 0.4% *w*/*v* solution of a high-molecular-weight branched PEI (M_w_ = 25,000 g mol^−1^, adjusted with 3% *w*/*v* NaCl) was poured onto the I-TFC membrane surface for 5 min, followed by rinsing with a 3% NaCl aqueous solution and subsequent curing at 90 °C for 20 min. The prepared PEI-grafted membranes were denoted as “I-TFC-g” and were stored in ultra-pure water until further testing and characterization (Figure 3).

### 2.3. Characterization

#### 2.3.1. Characterization of TFC Membranes

The surface chemical composition and the functional groups of the membranes were investigated by attenuated total reflection Fourier-transform infra-red (ATR-FTIR) spectroscopy and X-ray photoelectron spectroscopy (XPS). The ATR-FTIR spectra were collected on a Platinum ATR spectrometer (Bruker, Ettlingen, Germany) in the spectral range from 4000 to 400 cm^−1^ with a resolution of 4 cm^−1^. The XPS experiments were conducted in an ultra-high vacuum (UHV) system (with a background pressure in the range of 10^−11^–10^−7^ mbar) equipped with a SPECS Phoibos 100 1D-DLD hemispherical analyzer and a dual-anode Mg/Al X-ray source. The spectra were collected with a Mg Kα X-ray source (1253.6 eV) and the data were processed using Specs Lab Prodigy software. An unmonochromatized MgKα line at 1253.6 eV and an analyzer pass energy of 15 eV (giving a full width at half maximum (FWHM) of 0.85 eV for the Ag3d5/2 peak) were used.

The cross-sectional and surface morphology of the prepared membranes were studied using a field emission scanning electron microscope (FE-SEM, JSM-7000F, JOEL, Tokyo, Japan). All membrane samples were sputter-coated with gold to become electrically conductive before SEM examination. For cross-sectional imaging, the samples were freeze-fractured in liquid nitrogen. The surface morphology of the membranes was characterized by atomic force microscopy (AFM) using a Bruker Dimension ICON AFM equipped with a Controller VI, and a topographical analysis was performed. The operational mode was the tapping mode, and the scans were conducted with a scan size of 2 μm.

The wetting properties of the prepared membranes were characterized by water contact-angle measurements. In more detail, 10 μL of deionized water was pipetted onto the membrane surface and the contact angles were measured with the ImageJ software (version 1.54p 17), as described in the literature [49]. The standard deviation was calculated from two measurements for each membrane.

#### 2.3.2. Performance Characterization

**Figure 4:**
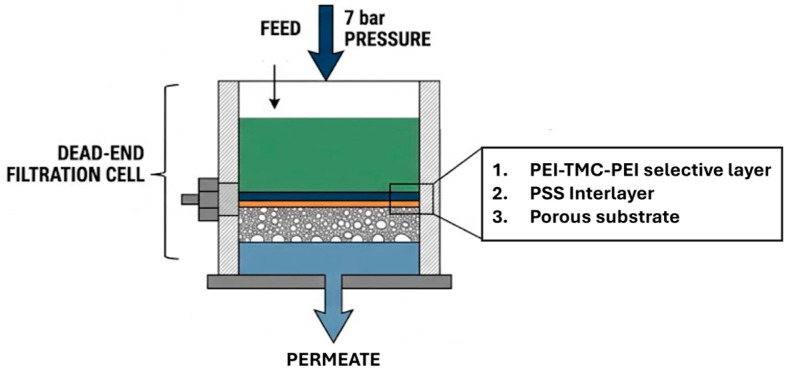
Schematic illustration of the dead-end membrane filtration test system.

The performance of the substrates and TFC membranes was evaluated through a dead-end high-pressure stirred cell (Sterlitech HP4750, Sterlitech, Auburn, Washington, USA) (Figure 4) connected to a nitrogen gas line for pressurization with an effective membrane surface area of 14.6 cm^2^. Prior to the measurements, the membranes were prepressed at 7 bar for 30 min to reach steady-state flux conditions. Pure-water flux measurements were conducted at room temperature under a constant stirring speed of 250 rpm at the same pressure.

The rejection performance of the membranes for various single salts was examined using feed solutions of MgSO_4_, Na_2_SO_4_, MgCl_2_ and NaCl, each with a 1000 ppm concentration, and the permeates were collected after reaching stable flux. The salt concentration determination was done with a bench conductivity meter. The pure-water permeability (PWP, L m^−2^ h^−1^ bar^−1^) and salt rejection (R, %) were calculated using Equations (1) and (2), respectively:

(1) $$PWP=\frac{\Delta V}{A_{m}\;\Delta t\;\Delta P}$$

(2) $$R=\left(1-\frac{C_{p}}{C_{f}}\right)\times 100$$

where ΔV is the permeate volume (L), Δt is the permeation time (h), A_m_ is the effective membrane area (m^2^), ΔP is the transmembrane pressure (bar), and C_p_ and C_f_ are the solute concentrations (ppm) at the permeate and feed, respectively. The pure-water permeability and salt rejection values are the average of two samples for each membrane type.

#### 2.3.3. Long-Term Stability Evaluation

To evaluate the long-term stability of the TFC membranes, a 1000 pm MgSO_4_ solution of the membranes was filtered (under 7 bar pressure) for 70 h. The flux (Equation (3)) and MgSO_4_ rejection were measured every 2.5 h. The MgSO_4_ feed solution was removed from the dead-end cell and replenished with a fresh one every time that half of the initial volume of the feed was filtered through the membrane.

(3) $$F=\frac{\Delta V}{A_{m}\;\Delta t}$$

where ΔV is the permeate volume (L), Δt is the permeation time (h), and A_m_ is the effective membrane area (m^2^).

## 3. Results and Discussion

### 3.1. Morphological Characterization

**Figure 5:**
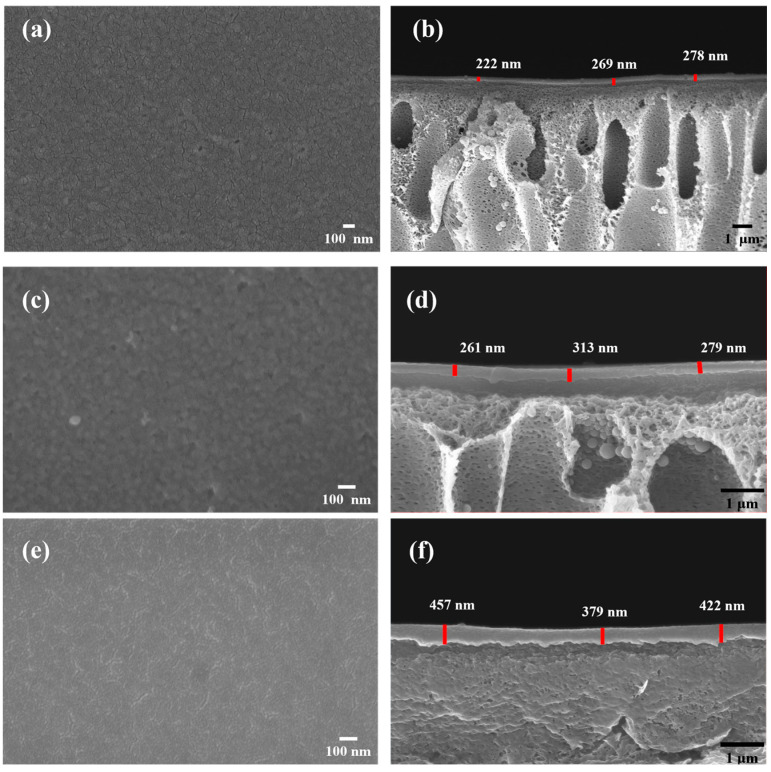
SEM images of the surface and cross-section of (**a**,**b**) the TFC control membrane, (**c**,**d**) the I-TFC membrane and (**e**,**f**) the I-TFC-g membrane.

The SEM and AFM analyses were conducted to characterize the surface morphology and thickness of the prepared membranes. The formation of small nano-aggregates (~20 nm) on the surface of the TFC and I-TFC membranes was observed because of the reaction kinetics of the IP reaction between PEI with TMC (Figure 5a,b). These were similar to those observed in previously reported PEI-TMC membranes [50,51]. Due to the long and branched molecular chains of PEI, its diffusion kinetics are slow compared to other amine monomers (e.g., piperazine); therefore, the size of the nanoaggregates on the PEI membrane surface was smaller than that observed for PIP-TMC membranes [52]. After PEI-grafting, nano-wrinkled structure formation was observed (Figure 5e).

As shown in Figure 5a–d (three positions of the PA layer were selected and the mean thickness of the PA layer was calculated), with the introduction of the PSS interlayer, the mean thickness was increased by 11% (from 256 nm to 284 nm). The PSS interlayer developed strong electrostatic interactions with PEI, thus resulting in increased PEI adsorption compared to the TFC membrane (without the PSS interlayer), and consequently, to a higher thickness. Another reason for the increased thickness could be the looser PA network attributed to the limited number of amino groups of PEI that reacted with TMC due to the strong electrostatic interactions between PSS and PEI. The subsequent PEI-grafting further led to increased thickness (419 nm) of the I-TFC-g membrane because of the reaction with residual acyl chloride groups of TMC and formation of a top PA layer.

**Figure 6:**
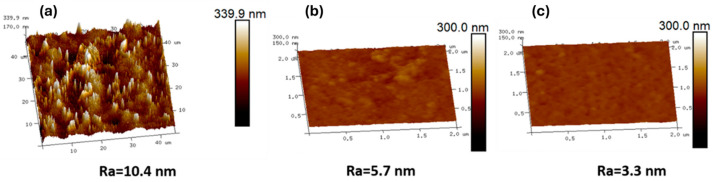
AFM analysis of the surface of (**a**) the TFC control membrane, (**b**) the I-TFC membrane and (**c**) the I-TFC-g membrane.

The AFM analysis showed that the control TFC membrane exhibited a rougher nano-nodule structure compared to I-TFC and I-TFC-g (Figure 6). The surface roughness after the incorporation of the PSS interlayer was significantly decreased from Ra = 10.4 nm (TFC) to 5.7 nm (I-TFC). The coating of the PSS interlayer via the LBL method led to the uniform coverage of the substrates’ surface with PEI macromolecules, which in turn facilitated the formation of a smooth PA layer. In addition, the retarded IP reaction as a consequence of the limited availability of PEI monomers to react with TMC due to their strong electrostatic interaction with PSS contributed to the formation of a smoother polyamide surface [31]. The subsequent PEI-grafting and thermal treatment at 90 °C for 20 min led to a further decrease in the roughness for the I-TFC-g membrane (from Ra = 5.7 nm to Ra = 3.3 nm). The smoother surface of the I-TFC membrane facilitated the grafting of PEI, resulting in a composite membrane with a more uniform surface charge distribution, which is crucial for enhancing the divalent ion separation selectivity and long-term stability.

### 3.2. Physicochemical Characterization

**Figure 7:**
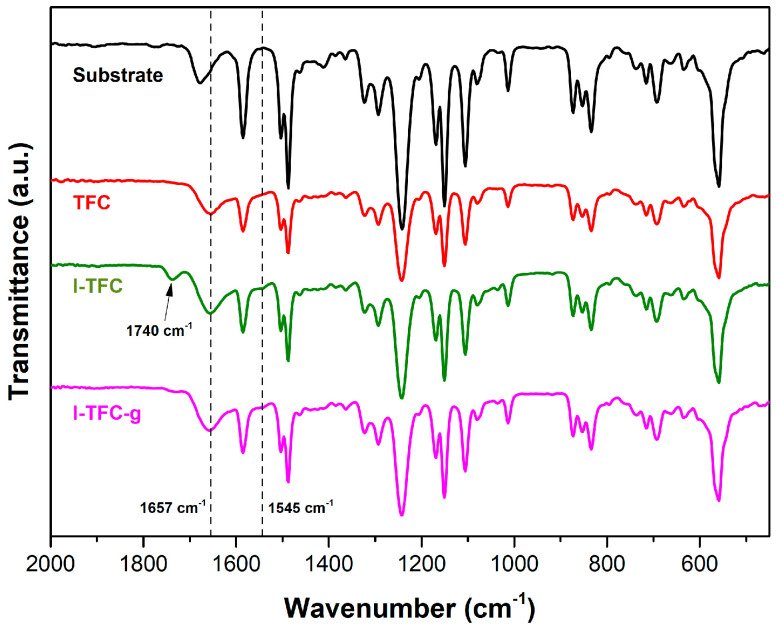
ATR-FTIR spectra of the S1 substrate, the TFC control membrane, the I-TFC membrane and the I-TFC-g membrane.

The chemical properties and compositions of the prepared NF membranes were examined using ATR-FTIR and XPS. When comparing the IR spectra of the S1 substrate and the control TFC membrane, a distinct absorption band at 1657 cm^−1^ and a shoulder at 1545 cm^−1^ were observed, corresponding to -N-C=O amide I and C–N/N–H (amide II coupling vibration), respectively (Figure 7). The presence of these characteristic peaks confirmed the successful construction of the PA active layer on the pristine S1 substrate. After the incorporation of the PSS interlayer, an additional peak was identified at 1740 cm^−1^, corresponding to the C=O vibration of the carboxylic acids that resulted from the hydrolysis of the many residual unreacted acyl chloride (O=C-Cl) groups of TMC [53,54], suggesting that the I-TFC membrane had many more -COOH groups compared to the control TFC membrane. Indeed, fewer amino groups of PEI were available to react with TMC due to their strong electrostatic interactions with the PSS interlayer; thus, many acyl chloride (O=C-Cl) groups of TMC remained unreacted and hydrolyzed to their carboxylic acid analogues. Following the PEI post-modification, the intensity of the former peak was significantly reduced, indicating the successful grafting reaction of the residual acyl chlorides with the amino groups of the PEI towards amide formation.

The XPS spectra exhibited five main peaks at binding energies of 168.4 eV, 199.0 eV, 285.15 eV, 400.1 eV and 532.44 eV, corresponding to S (2p), Cl (2p), C (1s), N (1s) and O (1s), respectively [55] (Figure S2). The elemental composition of the selective surface is presented in Table 1. The incorporation of the PSS interlayer resulted in a decreased nitrogen content and a significantly increased oxygen content (21.46%) compared to the TFC membrane. The latter was due to the carboxylic acid group increase, which originated from the unreacted acyl chlorides of TMC; this is consistent with the ATR-FTIR data.

Following the PEI post-modification, the surface of the I-TFC-g membrane showed a significantly increased nitrogen content of 8.48% compared to the corresponding membrane before grafting (6.37%), indicating that the grafting reaction can effectively increase the number of amino groups on the membrane surface, and therefore, its positive charge.

**Figure 8:**
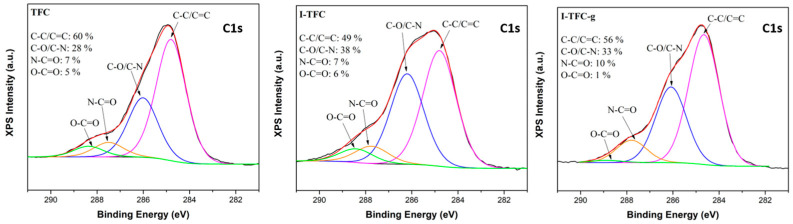
C1s core-level spectra of the prepared TFC, I-TFC and I-TFC-g membranes.

The high-resolution spectra of the XPS survey can be further used to elucidate the chemical bonds of the membrane surface. The C 1s core level can be deconvoluted to four peaks at 284.6 eV, 286.1 eV, 287.4 eV and 288.5 eV, ascribed to C=C/C-C, C-N/C-O, -N-C=O and O-C=O, respectively (Figure 8) [56].

The crosslinking degree of the polyamide layer can be estimated by the peak area ratio of the N-C=O to O=C-O groups [46]. Generally, a higher ratio of N-C=O to O=C-O groups indicates a higher degree of crosslinking in the polyamide layer. As shown in Table 1, the crosslinking degree and C 1s high-resolution XPS analysis suggest that the PA layer with a PSS interlayer had a slightly lower crosslinking degree and more carboxyl groups than the PA layer of the control membrane (the ratio of N-C=O to O=C-O was lower in I-TFC compared to the TFC membrane). The lower crosslinking degree was the direct consequence of the retarded IP reaction caused by the inhibition of PEI release by the PSS interlayer, as explained earlier. Therefore, many unreacted acyl chloride groups of TMC monomers were hydrolyzed into carboxyl groups, contributing to the enhancement of the O-C=O content, and thus, to the slight decrease in the crosslinking degree.

After PEI-grafting, the percentage of O=C-N amide bonds (peak at 287.4 eV) increased (from 7 to 10%), which was attributed to the new amide bond formation, further confirming the successful grafting reaction of PEI on the I-TFC-g membrane surface. In addition, a significant increase in the crosslinking degree of the PA network from 54% to 90% was observed (Table 1). On the contrary, the carboxylic acid content formed by the hydrolysis of unreacted -COCl sharply decreased from 6 to 1%, due to the successful grafting reaction of PEI with the remaining acyl chlorides. The latter was also verified by the disappearance of the peak at 1740 cm^−1^ corresponding to carboxylic acid groups (Figure 7).

In the N 1 s spectra (Figure S3), the nitrogen species were identified in three distinct states, including –NH_2_ at 399.4 eV, N-C=O at 400.4 eV, and –NH_3_^+^ at 401.8 eV, respectively [57]. A higher percentage of –NH_3_^+^ groups was observed for the I-TFC-g membrane, suggesting that PEI-grafting increased both the positive charge and the compactness of the selective layer, which enhanced ion-sieving and Donnan exclusion, and consequently contributed to improving the divalent/monovalent separation selectivity.

**Figure 9:**
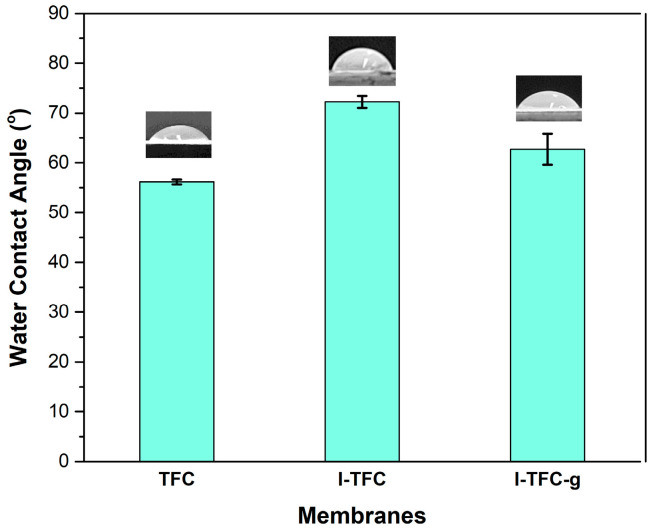
WCA measurements of TFC, I-TFC and I-TFC-g membranes.

The hydrophilicity of the membrane surface was studied by the water contact angle (WCA). The introduction of a PSS layer led to an increased contact angle for the I-TFC membrane (Figure 9), probably associated with its reduced roughness, in agreement with the well-established “hydrophilicity–roughness” relationship as described by the Wenzel equation [58]. Following PEI-grafting, the contact angle value was reduced, suggesting a more hydrophilic membrane surface (from 72% for I-TFC to 63% for I-TFC-g). This can probably be ascribed to the increased number of hydrophilic amino groups (-NH_3_^+^) of the grafted PEI.

### 3.3. Nanofiltration Performance

**Figure 10:**
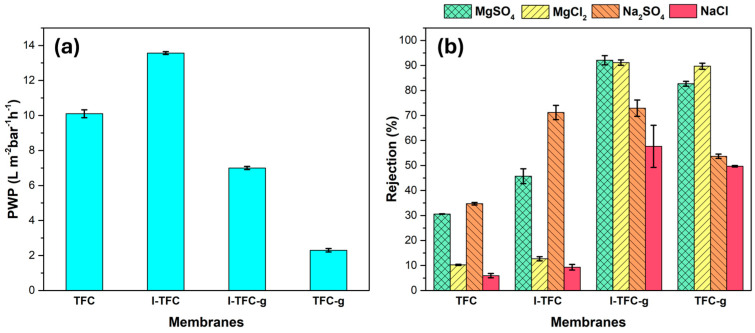
PWP (**a**) and salt rejections (**b**) of the prepared TFC membranes.

The incorporation of the polyelectrolyte PSS as an interlayer significantly enhanced the PWP without compromising the salt selectivity (Figure 10a). Specifically, although I-TFC had a higher selective layer thickness, it exhibited a PWP value of ~13.5 L m^−2^ h^−1^ bar^−1^, which was 1.3 times higher than that of the control membrane (~10 L m^−2^ h^−1^ bar^−1^ for TFC). This improvement can be mainly attributed to the continuous PSS interlayer, which prevented the PA polymer from intruding into and blocking the substrate pores and accelerated water transport (gutter effect) due to its hydrophilic nature. Another reason for this improvement could be the looser PA network of the I-TFC membrane.

The introduction of the PSS interlayer also resulted in a significant rejection increase for MgSO_4_ and Na_2_SO_4_ (from 31 to 46 ± 3% and from 35 to 71 ± 3%, respectively; Figure 10b). This was due to the negative surface charge of the I-TFC membrane, stemming from the negatively charged PSS interlayer and the increased concentration of carboxylic acid groups formed from the hydrolysis of residual -COCl groups, which facilitated divalent anion SO_4_^−2^ repulsion, according to the Donnan exclusion mechanism.

The effect of PEI-grafting on the separation performance was also investigated. After PEI-grafting, the PWP value was reduced from ~13.5 L m^−2^ h^−1^ bar^−1^ for I-TFC to ~7 L m^−2^ h^−1^ bar^−1^ for I-TFC-g, suggesting that the increased crosslinking degree and enhanced thickness of the PA layer had a negative effect on the water permeability. In addition, the I-TFC-g membrane demonstrated substantially improved rejections for MgSO_4_, MgCl_2_, and NaCl compared to the I-TFC membrane (Figure 10b). The higher rejections mainly resulted from the enhancement of the surface positive charge and the crosslinking degree, as evidenced by the XPS measurements, which simultaneously reinforced the size-sieving and Donnan exclusion mechanisms. Indeed, the rejection for MgSO_4_ increased from 46 ± 3% for I-TFC to 92 ± 2% for I-TFC-g, while a nine-times-higher salt rejection was observed for MgCl_2_ from 13 ± 1 to 91 ± 1 (Figure 10b). On the contrary, the rejection for Na_2_SO_4_ remained almost unchanged after PEI-grafting (73 ± 3%). The higher increment in Mg^2+^ rejections can be explained by the surface charge inversion from negative in the I-TFC membrane (from the carboxylic acids), which attracted Mg^2+^, to positive upon PEI-grafting, which enhanced the Mg^2+^ repulsions, and therefore, their rejection (Donnan exclusion). For Na_2_SO_4_, despite the higher attraction of SO_4_^−2^ to the positive surface, no rejection decrease was observed, attributed to the increased crosslinking degree of the PA network and probably to the negative PSS interlayer beneath the PA [41,42,43].

In addition, for comparison reasons, a TFC membrane (without an interlayer) grafted with PEI (TFC-g) was prepared to study the NF performance. The TFC-g membrane demonstrated a significantly decreased PWP value (2.5 L m^−2^ h^−1^ bar^−1^) compared to the I-TFC-g membrane. This evidence suggests that the incorporation of the PSS interlayer led to a water permeability enhancement of 180%. On the other hand, the TFC-g membrane displayed high salt rejections for MgSO_4_ and MgCl_2_ (83% and 90%, respectively; Figure 10b). This implies that PEI-grafting onto the TFC surface alone is sufficient to achieve very high salt rejections for MgSO_4_ and MgCl_2_, close to the ones obtained after PEI-grafting on a PSS-modified TFC membrane (91–92%), highlighting the dominant effect of grafting on the separation performance. To conclude, grafting with PEI endowed the membrane surface with an increased compactness and positive charge, thus reinforcing both the size-sieving and the Donnan exclusion mechanisms, while the incorporation of the hydrophilic PSS interlayer contributed to a water permeability enhancement.

As shown in Table S1, the performance of the I-TFC-g membrane was compared to other NF membranes for di/monovalent ion separation in the literature [30,59,60,61,62,63,64,65,66,67]. The prepared membrane in this work showed a similar or even higher water permeability and comparable salt rejections, highlighting its potential for this application.

### 3.4. Long-Term Stability

**Figure 11:**
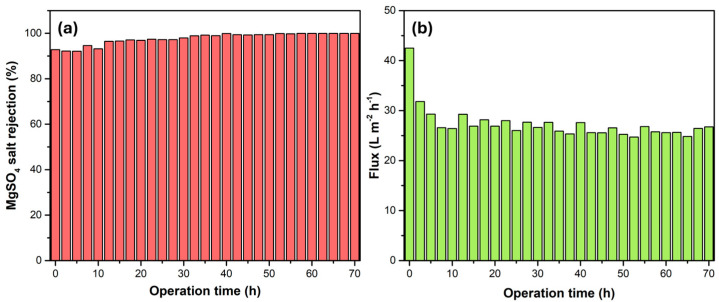
Long-term stability performance using MgSO_4_ salt rejection (**a**) and flux (**b**) of I-TFC-g membrane.

Stability is a key factor in evaluating the efficiency of NF membranes. A 1000 ppm MgSO_4_ solution served as a feed solution to evaluate the stability of the I-TFC-g membrane (Figure 11). During the 70 h continuous operation, the flux experienced an initial drop (from 42.5 to 26.8 Lm^−2^ h^−1^ bar^−1^) followed by a stable performance. This initial reduction in the water flux was primarily attributed to the slow compaction of the porous support, as reported for NF membranes [68]. After ~15 h of operation, the opposite trend was observed for the MgSO_4_ rejection (increased from 92% to 99%), which can be ascribed to membrane fouling, due to precipitation of MgSO_4_ salt, which led to pore clogging. Nevertheless, the subsequent good stability of the I-TFC-g membrane underscores its promising potential for applications.

## 4. Conclusions

In this study, the preparation of a triple-layered NF membrane consisting of a negatively charged PSS interlayer, a PA layer and a top PEI-grafted layer with an enhanced separation performance is reported. In more detail, the PSS interlayer incorporation resulted in a significantly smoother surface, endowing the I-TFC membrane with increased salt rejections (from 31% MgSO_4_ and 35% Na_2_SO_4_ for the TFC membrane to 46% and 71% for I-TFC, respectively), and an enhanced water permeability (from 10 L m^−2^ h^−1^ bar^−1^ to 13.5 L m^−2^ h^−1^ bar^−1^) compared to the control membrane. Following PEI-grafting, the resulting I-TFC-g membrane displayed much higher salt rejections due to its higher crosslinking degree and increased surface positive charge. In addition, the I-TFC-g membrane exhibited simultaneously higher rejections (92% MgSO_4_, 91% MgCl_2_, 73% Na_2_SO_4_) and an increased water permeability (7 L m^−2^ h^−1^ bar^−1^) when compared with the corresponding PEI-grafted membrane, which did not possess the PSS interlayer (83% MgSO_4_, 90% MgCl_2_, 54% Na_2_SO_4_ and 2.5 L m^−2^ h^−1^bar^−1^). Therefore, the incorporation of the PSS interlayer along with PEI surface-grafting could be used to combat the permeability–selectivity trade-off and improve the NF performance.

## Figures and Tables

**Table 1:** Relative atomic concentration of carbon, oxygen, nitrogen, sulfur and chlorine as derived from the XPS analysis (±0.05).

Membrane	C (%)	O (%)	N (%)	S (%)	Cl (%)	Crosslinking Degree (%)
TFC	76.06	14.42	8.25	1.27	-	58
I-TFC	70.63	21.46	6.37	1.11	0.42	54
I-TFC-g	71.68	16.54	8.48	2.25	1.06	90

## Data Availability

The original contributions presented in this study are included in the article/Supplementary Materials. Further inquiries can be directed to the corresponding author.

## Supplementary Materials

The following supporting information can be downloaded at: https://www.mdpi.com/article/10.3390/polym18101242/s1, Figure S1: FTIR spectra of the substrate membrane S1, neat PEI and control substrate without containing PEI; Figure S2: XPS survey spectra of the prepared membranes; Figure S3: Deconvolution of the XPS N 1s peak for the membranes TFC, I-TFC and I-TFC-g; Table S1: Comparison of the performance of NF membranes reported in the literature with the prepared membrane in this work.

## References

[1] Boretti A., Rosa L. (2019). Reassessing the projections of the World Water Development Report. npj Clean Water.

[2] European Environment Agency (2024). Europe’s State of Water 2024. The Need for Improved Water Resilience.

[3] Toreti A., Bavera D., Acosta N.J., Arias-Muñoz C., Avanzi F., Marinho F.B.P., De J.A., Di C.C., Ferraris L., Fioravanti G. (2023). Drought in Europe March 2023.

[4] Košutić K., Furač L., Sipos L., Kunst B. (2005). Removal of arsenic and pesticides from drinking water by nanofiltration membranes. Sep. Purif. Technol..

[5] Gozálvez-Zafrilla J., Sanz-Escribano D., Lora-García J., Hidalgo M.L. (2008). Nanofiltration of secondary effluent for wastewater reuse in the textile industry. Desalination.

[6] Majamaa K., Warczok J., Lehtinen M. (2011). Recent operational experiences of FILMTEC™ NF270 membrane in Europe. Water Sci. Technol..

[7] Boo C., Wang Y., Zucker I., Choo Y., Osuji C.O., Elimelech M. (2018). High Performance Nanofiltration Membrane for Effective Removal of Perfluoroalkyl Substances at High Water Recovery. Environ. Sci. Technol..

[8] Guo S., Wan Y., Chen X., Luo J. (2021). Loose nanofiltration membrane custom-tailored for resource recovery. Chem. Eng. J..

[9] Pu L., Xia Q., Wang Y., Bu Y., Zhang Q., Gao G. (2022). Tailored nanofiltration membranes with enhanced permeability and antifouling performance towards leachate treatment. J. Membr. Sci..

[10] Ismail A.F., Jye L.W. (2017). Nanofiltration Membranes: Synthesis, Characterization, and Applications.

[11] Wang K., Wang X., Januszewski B., Liu Y., Li D., Fu R., Elimelech M., Huang X. (2022). Tailored design of nanofiltration membranes for water treatment based on synthesis–property–performance relationships. Chem. Soc. Rev..

[12] Lim Y.J., Goh K., Nadzri N., Wang R. (2024). Thin-film composite (TFC) membranes for sustainable desalination and water reuse: A perspective. Desalination.

[13] Lim Y.J., Goh K., Wang R. (2022). The coming of age of water channels for separation membranes: From biological to biomimetic to synthetic. Chem. Soc. Rev..

[14] Liu M., Yao G., Cheng Q., Ma M., Yu S., Gao C. (2012). Acid stable thin-film composite membrane for nanofiltration prepared from naphthalene-1,3,6-trisulfonylchloride (NTSC) and piperazine (PIP). J. Membr. Sci..

[15] Li X., Zhang C., Zhang S., Li J., He B., Cui Z. (2015). Preparation and characterization of positively charged polyamide composite nanofiltration hollow fiber membrane for lithium and magnesium separation. Desalination.

[16] Kong C., Kanezashi M., Yamomoto T., Shintani T., Tsuru T. (2010). Controlled synthesis of high performance polyamide membrane with thin dense layer for water desalination. J. Membr. Sci..

[17] Kim S.H., Kwak S.-Y., Suzuki T. (2005). Positron Annihilation Spectroscopic Evidence to Demonstrate the Flux-Enhancement Mechanism in Morphology-Controlled Thin-Film-Composite (TFC) Membrane. Environ. Sci. Technol..

[18] Peng Q., Wang R., Zhao Z., Lin S., Liu Y., Dong D., Wang Z., He Y., Zhu Y., Jin J. (2024). Extreme Li-Mg selectivity via precise ion size differentiation of polyamide membrane. Nat. Commun..

[19] Liang Y., Zhu Y., Liu C., Lee K.-R., Hung W.-S., Wang Z., Li Y., Elimelech M., Jin J., Lin S. (2020). Polyamide nanofiltration membrane with highly uniform sub-nanometre pores for sub-1 Å precision separation. Nat. Commun..

[20] Panagiotou F., Zuburtikudis I., Abu Khalifeh H., Nashef E., Deimede V. (2025). GO and surfactant assisted regulation of polyamide nanofiltration membranes for improved separation performance. Sep. Purif. Technol..

[21] Wang Y., Zhang T., Shen K., Wang D., Wang X. (2024). Low temperature regulated reverse interfacial polymerization for fabricating thin film composite membranes based on nanofibrous substrates. Colloids Surf. A Physicochem. Eng. Asp..

[22] Werber J.R., Osuji C.O., Elimelech M. (2016). Materials for next-generation desalination and water purification membranes. Nat. Rev. Mater..

[23] Yang Z., Guo H., Tang C.Y. (2019). The upper bound of thin-film composite (TFC) polyamide membranes for desalination. J. Membr. Sci..

[24] Wu C., Long L., Yang Z., Hu Y., Peng L.E., Tang C.Y. (2025). Unraveling the role of funnel effect vs. gutter effect in water permeance and antifouling performance of polyamide nanofiltration membranes. Water Res..

[25] Yang Z., Sun P.-F., Li X., Gan B., Wang L., Song X., Park H.-D., Tang C.Y. (2020). A Critical Review on Thin-Film Nanocomposite Membranes with Interlayered Structure: Mechanisms, Recent Developments, and Environmental Applications. Environ. Sci. Technol..

[26] Gao S., Zhu Y., Gong Y., Wang Z., Fang W., Jin J. (2019). Ultrathin Polyamide Nanofiltration Membrane Fabricated on Brush-Painted Single-Walled Carbon Nanotube Network Support for Ion Sieving. ACS Nano.

[27] Zhu X., Cheng X., Luo X., Liu Y., Xu D., Tang X., Gan Z., Yang L., Li G., Liang H. (2020). Ultrathin Thin-Film Composite Polyamide Membranes Constructed on Hydrophilic Poly(vinyl alcohol) Decorated Support Toward Enhanced Nanofiltration Performance. Environ. Sci. Technol..

[28] Dai R., Li J., Wang Z. (2020). Constructing interlayer to tailor structure and performance of thin-film composite polyamide membranes: A review. Adv. Colloid Interface Sci..

[29] Wang X., Gao N., Wang L., Liao Y. (2023). Polyelectrolyte interlayer assisted interfacial polymerization fabrication of a dual-charged composite nanofiltration membrane on ceramic substrate with high performance. J. Membr. Sci..

[30] Tian R., Zhang H., Wang J., Dilxat D., Xie T., Qi Q., Wang Y. (2024). Nanofiltration membrane functionalization with enhanced hardness cation removal using a mono-component interlayer. Desalination.

[31] He T., Chen F. (2024). Enhanced separation performance of composite nanofiltration membranes via electrostatic air spray PSS/PEI interlayer. Desalination.

[32] Hu P., Tian B., Xu Z., Niu Q.J. (2020). Fabrication of high performance nanofiltration membrane on a coordination-driven assembled interlayer for water purification. Sep. Purif. Technol..

[33] Burts K.S., Plisko T.V., Davydova M.V., Makarava M.S., Yuan B., Penkova A.V., Ermakov S.S., Bildyukevich A.V. (2025). The effect of polydiallyldimethylammonium chloride molecular weight in the intermediate layer on the structure and performance of thin film composite membranes for nanofiltration prepared via interfacial polymerization. Colloids Surf. A Physicochem. Eng. Asp..

[34] Yang Z., Wang F., Guo H., Peng L.E., Ma X.-H., Song X.-X., Wang Z., Tang C.Y. (2020). Mechanistic Insights into the Role of Polydopamine Interlayer toward Improved Separation Performance of Polyamide Nanofiltration Membranes. Environ. Sci. Technol..

[35] Hu M., Li X., Tao R., Mai Z., Chen X., Gui S., Matsuyama H., Li J. (2023). Highly permeable polyamide nanofiltration membranes with crumpled structures regulated by polydopamine-piperazine-halloysite interlayer. Desalination.

[36] Al-Nahari A., Wang E., Zhang Q., Wu W., Ali A., Zhang Q., Su B. (2025). Enhancing thermal stability of nanofiltration membrane with polydopamine/melamine co-deposition interlayer. J. Membr. Sci..

[37] Zhu X., Yang Z., Gan Z., Cheng X., Tang X., Luo X., Xu D., Li G., Liang H. (2020). Toward tailoring nanofiltration performance of thin-film composite membranes: Novel insights into the role of poly(vinyl alcohol) coating positions. J. Membr. Sci..

[38] Tang Y.-J., Cheng L., Wu T., Xu Z.-L. (2024). Quantitative analysis of the influence of PVA interlayer on the permeation resistance of TFC NF membranes. Desalination.

[39] Zhao S., Xue S., Li L., Ji C., Li P., Niu Q.J. (2023). A comprehensive evaluation of PVA enhanced polyamide nanofiltration membranes: Additive versus interlayer. Colloids Surf. A Physicochem. Eng. Asp..

[40] Gu J.E., Lee S., Stafford C.M., Lee J.S., Choi W., Kim B.Y., Baek K.Y., Chan E.P., Chung J.Y., Bang J. (2013). Molecular layer-by-layer assembled thin-film composite membranes for water desalination. Adv. Mater..

[41] Guo C., Li N., Qian X., Shi J., Jing M., Teng K., Xu Z. (2020). Ultra-thin double Janus nanofiltration membrane for separation of Li^+^ and Mg^2+^: “Drag” effect from carboxyl-containing negative interlayer. Sep. Purif. Technol..

[42] Zhao B., Pei M., Guo X., Zhang Y., Wang L., Zhang Z. (2025). Enhanced Mg^2+^/Li^+^ separation by Janus nanofiltration membrane incorporated with negatively-charged COF interlayer. Sep. Purif. Technol..

[43] Yang H., Hou J., Chen V., Xu Z. (2016). Janus Membranes: Exploring Duality for Advanced Separation. Angew. Chem. Int. Ed..

[44] Zhang L., Hu M., Matsuyama H., Li X. (2024). Preparation strategies of the positively charged nanofiltration membrane: A comprehensive review. Sep. Purif. Technol..

[45] Liu Y., Matsuyama H., Liu Y., Li Y., Zheng J., Dai Z., Van der Bruggen B. (2025). Advances in positively charged nanofiltration membranes: Targeted strategies for optimized water treatment. Desalination.

[46] Wang J., Zhang H., Tian R., Shen H., Li W.-H., Wang Y. (2024). Enhancing Mg^2+^/Li^+^ separation performance of nanofiltration membranes through polyelectrolyte modulation and surface modification. J. Membr. Sci..

[47] Cheng X., Zhang Y., Shao S., Lai C., Wu D., Xu J., Luo X., Xu D., Liang H., Zhu X. (2023). Highly permeable positively charged nanofiltration membranes with multilayer structures for multiple heavy metal removals. Desalination.

[48] Choi W., Jeon S., Kwon S.J., Park H., Park Y.-I., Nam S.-E., Lee P.S., Lee J.S., Choi J., Hong S. (2017). Thin film composite reverse osmosis membranes prepared via layered interfacial polymerization. J. Membr. Sci..

[49] Tzoumani I., Druvari D., Andrikopoulos K.C., Dominguez-Alfaro A., Malliaras G.G., Kallitsis J.K. (2024). Facile fabrication of dual-conductivity, humidity-responsive single-layer membranes: Towards advanced applications in sensing, actuation, and energy generation. J. Mater. Chem. C.

[50] Zhang H.-Z., Sun J.-Y., Zhang Z.-L., Xu Z.-L. (2021). Hybridly charged NF membranes with MOF incorporated for removing low-concentration surfactants. Sep. Purif. Technol..

[51] Xu Y., Peng H., Luo H., Zhang Q., Liu Z., Zhao Q. (2022). High performance Mg^2+^/Li^+^ separation membranes modified by a bis-quaternary ammonium salt. Desalination.

[52] Cheng X., Qin Y., Ye Y., Chen X., Wang K., Zhang Y., Figoli A., Drioli E. (2021). Finely tailored pore structure of polyamide nanofiltration membranes for highly-efficient application in water treatment. Chem. Eng. J..

[53] Wu D., Huang Y., Yu S., Lawless D., Feng X. (2014). Thin film composite nanofiltration membranes assembled layer-by-layer via interfacial polymerization from polyethylenimine and trimesoyl chloride. J. Membr. Sci..

[54] Zimudzi T.J., Feldman K.E., Sturnfield J.F., Roy A., Hickner M.A., Stafford C.M. (2018). Quantifying Carboxylic Acid Concentration in Model Polyamide Desalination Membranes via Fourier Transform Infrared Spectroscopy. Macromolecules.

[55] Xu P., Wang W., Qian X., Wang H., Guo C., Li N., Xu Z., Teng K., Wang Z. (2019). Positive charged PEI-TMC composite nanofiltration membrane for separation of Li^+^ and Mg^2+^ from brine with high Mg^2+^/Li^+^ ratio. Desalination.

[56] Qian Y., Wu H., Sun S.-P., Xing W. (2020). Perfluoro-functionalized polyethyleneimine that enhances antifouling property of nanofiltration membranes. J. Membr. Sci..

[57] Xie T., Xu Z., Li F., Li P., Sun H., Niu Q.J. (2025). Sandwich-structured nanofiltration membrane for efficient magnesium-lithium separation. Sep. Purif. Technol..

[58] Wenzel R.N. (1936). Resistance of solid surfaces to wetting by water. Ind. Eng. Chem..

[59] Li G., Han J., Li S., Zhao L., Zhang G., Zheng S., Lv D., Wang F., Deng H. (2026). Dually charged quaternized PEI/lignin-polyamide composite nanofiltration membrane toward highly efficient di-/monovalent ion separation. J. Membr. Sci..

[60] Cheng Y., Sun Y., Dong S., Ma L., He X., Xu Q., Wang Z., Tang Y., Li P., Hai C. (2026). Construction of an ultrathin separation layer of PEI-TMC NF membrane via a retarder for high-efficiency Li^+^ / Mg^2+^ separation. Sep. Purif. Technol..

[61] Shao W., Liu C., Ma H., Hong Z., Xie Q., Lu Y. (2019). Fabrication of pH-sensitive thin-film nanocomposite nanofiltration membranes with enhanced performance by incorporating amine-functionalized graphene oxide. Appl. Surf. Sci..

[62] Wang Q., Dong Y., Ma J., Wang H., Xue X., Bai C., Lin M., Luo L., Gao C., Xue L. (2023). Polyamide/polyethylene thin film composite (PA/PE-TFC) NF membranes prepared from reverse-phase interface polymerization (RIP) for improved Mg(II)/Li(I) separation. Desalination.

[63] Wu M.-B., Ye H., Zhu Z.-Y., Chen G.-T., Ma L.-L., Liu S.-C., Liu L., Yao J., Xu Z.-K. (2022). Positively-charged nanofiltration membranes constructed via gas/liquid interfacial polymerization for Mg^2+^/Li^+^ separation. J. Membr. Sci..

[64] Gu T., Zhang R., Zhang S., Shi B., Zhao J., Wang Z., Long M., Wang G., Qiu T., Jiang Z. (2022). Quaternary ammonium engineered polyamide membrane with high positive charge density for efficient Li^+^/Mg^2+^separation. J. Membr. Sci..

[65] Bi Q., Zhang C., Liu J., Liu X., Xu S. (2021). Positively charged zwitterion-carbon nitride functionalized nanofiltration membranes with excellent separation performance of Mg^2+^/Li^+^ and good antifouling properties. Sep. Purif. Technol..

[66] Wu H., Lin Y., Feng W., Liu T., Wang L., Yao H., Wang X. (2020). A novel nanofiltration membrane with [MimAP][Tf2N] ionic liquid for utilization of lithium from brines with high Mg^2+^/Li^+^ ratio. J. Membr. Sci..

[67] Fang S., Guan K., Zhou S., Song Q., Shi Y., Fu W., Li Z., Xu P., Hu M., Mai Z. (2024). Ternary-coordination-regulated polyamide nanofiltration membranes for Li^+^/Mg^2+^ separation. Desalination.

[68] Aghajani M., Wang M., Cox L.M., Killgore J.P., Greenberg A.R., Ding Y. (2018). Influence of support-layer deformation on the intrinsic resistance of thin film composite membranes. J. Membr. Sci..

