# Supplementary material for: Enhancing Separation Performance of PA Nanofiltration Membrane Through Polyelectrolyte PSS Interlayer and Surface Modification
Source: Polymers (Basel). 2026 May 19;18(10):1242. doi: 10.3390/polym18101242 (PMC13210598; doi:10.3390/polym18101242)
Supplement: Supplementary file 1 [file polymers-18-01242-s001.zip › polymers-4296681-supplementary.pdf]

SUPPLEMENTARY INFORMATION

Enhancing separation performance of PA nanofiltration membrane through polyelectrolyte PSS interlayer and surface modification

Fotios Panagiotou[a], Georgia Zafeiropoulou[a], Franceska Gojda[b,c], Kiriaki Chrissopoulou[b], Ioannis Zuburtikudis[d], Valadoula Deimede[a,]*

[a] *Department of Chemistry, University of Patras, GR-26504, Patras, Greece*

[b] *Institute of Electronic Structure and Laser, Foundation for Research and Technology - Hellas, P. O. Box 1527, 711 10 Heraklion Crete, Greece*

[c] *Department of Physics, University of Crete, GR-70013 Heraklion, Greece*

[d] *Chemical Engineering Department, Abu Dhabi University, Abu Dhabi 59911, United Arab Emirates*

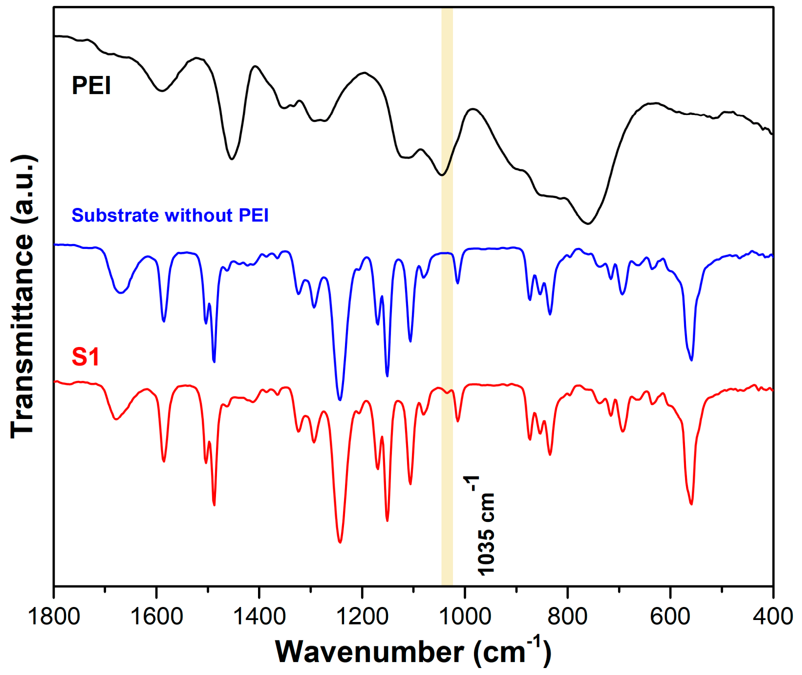

Figure S1. FTIR spectra of the substrate membrane S1, neat PEI and control substrate without containing PEI.

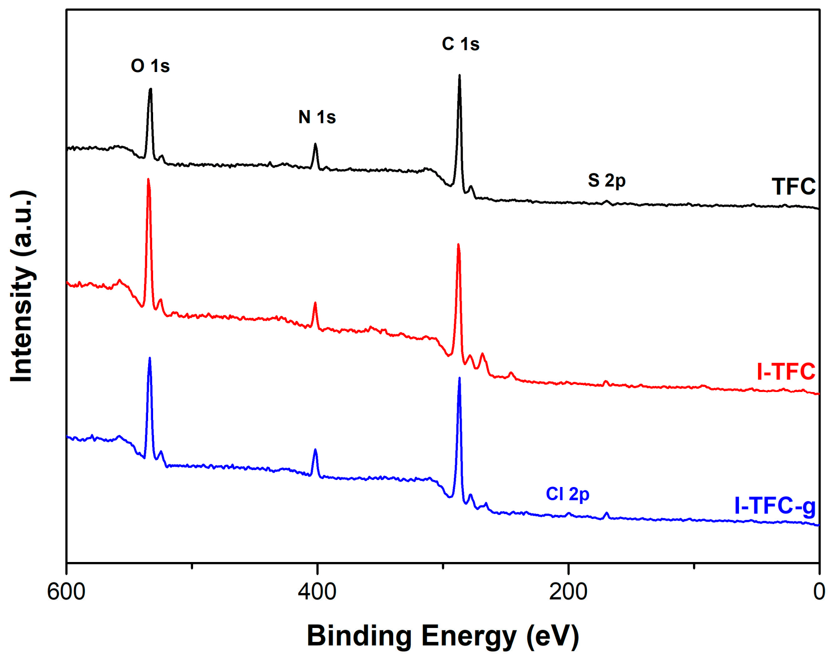

Figure S2. XPS survey spectra of the prepared membranes.

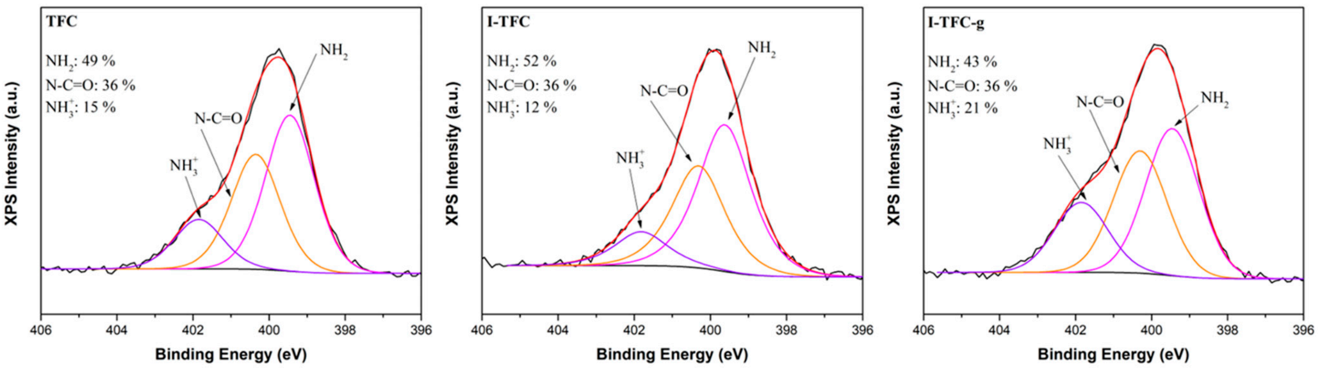

Figure S3. Deconvolution of the XPS N 1s peak for the membranes TFC, I-TFC and I-TFC-g.

Table S1. Comparison of the performance of NF membranes reported in the literature with the prepared membrane in this work.

Membrane	PWP	$R(MgSO_4)$	$R(MgCl_2)$	$R(Na_2SO_4)$	R(NaCl)	Ref.
	$Lm^{-2}h^{-1}bar^{-1}$					
QPEI/PAL NF	11.0	~98%	98.4 %	92.6 %	~40%	[59]
PEI/$SC_{0.075}$-TMC	~18	~85%	~98%	~38%	~55%	[60]
TFN-GO-PEI-40	12.4	97.8%	93.4%	98.2%	38.2%	[61]
TMC/PIP(RIP)	4.8	96.7%	94.3%	~96%	~35%	[62]
GLIP	10	~85%	98.5%	~62%	~37%	[63]
PEI/TMC/DHTAB	~6	~98%	99.2%	72%	~73%	[64]
MBCN/TMC	5.6	~95%	97.4%	~43%	~52%	[65]
NF-IL-2.0%	6.3	89.0%	83.8%	54.6%	30.1%	[66]
TA/PEI-Cu/TMC	4.87	~98%	95.9%	77%	38%	[67]
Alg. membrane	7.23	-	~91%	~98%	~40%	[30]
**I-TFC-g**	**7**	**92%**	**91%**	**73%**	**58%**	**This work**
